# Reviewer acknowledgement 2013

**DOI:** 10.1186/1471-2105-15-67

**Published:** 2014-03-11

**Authors:** Irene Pala

**Affiliations:** 1 BioMed Central, 236 Gray’s Inn, Road, London WC1X 8HB, UK

## Abstract

**Contributing reviewers:**

The editors of BMC Bioinformatics would like to thank all our reviewers who have contributed their time to the journal in Volume 14 (2013).

## 

Hidenao Abe

Japan

Mohamed Abouelhoda

Egypt

Jörg Ackermann

Germany

George Acquaah-Mensah

USA

Alex Adai

USA

Pankaj Agarwal

USA

Shandar Ahmad

Japan

Tero Aittokallio

Finland

Martin Akerman

USA

Mete Akgün

Turkey

Most M Akhtar

Italy

Tatsuya Akutsu

Japan

Raul Alcaraz Martinez

Spain

Max Alekseyev

USA

Dimitra Alexopoulou

Germany

Eivind Almaas

Norway

Uri Alon

Israel

Mohammed Alquraishi

USA

Fernando Amat

USA

Nicola Ancona

Italy

Timothy Andersen

USA

Bill Andreopoulos

Germany

Simon Andrews

UK

Peter Antal

Hungary

Maciek Antoniewicz

USA

Emilia Apostolova

USA

Sivaram Arabandi

USA

Kellie Archer

USA

Cecilia Arighi

USA

Masanori Arita

Japan

Martin Aryee

USA

Kiyoshi Asai

Japan

Marcella Attimonelli

Italy

Raymond Auerbach

USA

Frederick Ausubel

USA

Dmitry Avtonomov

USA

Apostolos Axenopoulos

Greece

Yesim Aydin Son

Turkey

Vasco Azevedo

Brazil

Mohan Babu

Canada

Michael Bada

USA

Guy Baele

Belgium

Pantelis Bagos

Greece

Timothy Bailey

Australia

Marc Bailly-Bechet

France

Eric Bair

USA

Tom Bair

USA

Javier Bajo

Spain

Erich Baker

USA

Rajib Bandopadhyay

India

Samprit Banerjee

USA

Vikas Bansal

USA

Zhirong Bao

USA

Nuno L Barbosa-Morais

Portugal

Anais Bardet

Switzerland

Debmalya Barh

India

Erhan Bas

USA

Katia Basso

USA

Saonli Basu

USA

Subhadip Basu

India

Ulrich Baumann

Germany

Niko Beerenwinkel

Switzerland

Jonas Behr

Switzerland

Robert Beiko

Canada

Stephan Beisken

UK

Tim Beissbarth

Germany

Jordi Benach

USA

Andrea Benazzo

Italy

Asa Ben-Hur

USA

Stephen Benz

USA

Casey Bergman

UK

Benjamin Berman

USA

Stephan Bernhart

Austria

Philippe Bessières

France

Jong Bhak

Korea South

Sandeep Bhat

USA

David Bickel

Canada

Joanna Biernacka

USA

Holly Bik

USA

Cemal Cagatay Bilgin

USA

Harald Binder

Germany

Pierre-Alain Binz

Switzerland

Tom Blackwell

USA

Daniel Blankenberg

USA

Marta Bleda

Spain

Alexander Bockmayr

Germany

Neio Boechat

Brazil

Marina Bogomolov

Israel

Jason Bohland

USA

Dan Bolser

UK

Alexander Bolshoy

Israel

Stefan Bonn

Germany

Eric Bonnet

France

Andrew Bordner

USA

Mark Borodovsky

USA

Lubor Borsig

Switzerland

Thomas Boudier

France

Anne-Laure Boulesteix

Germany

Paul Boutros

Canada

John Boyle

USA

Serdar Bozdag

USA

Ivana Bozic

USA

Pascal Braun

Germany

Nicolas Bray

USA

Laurent Brehelin

France

Rainer Breitling

UK

Rainer Breitling

Netherlands

Alessandro Bria

Italy

Ryan Brinkman

Canada

Mitchell Brittnacher

USA

David Broadhurst

Canada

Yana Bromberg

USA

C. Titus Brown

USA

James Bruce

USA

Kim Brugger

UK

Michal Brylinski

USA

Daniel Buchan

UK

Josh Buckner

USA

Teresia Buza

USA

Rongman Cai

USA

Weidong Cai

Australia

Yudong Cai

China

Scott Cain

USA

Raffaele Calogero

Italy

Stefano Calza

Italy

Sander Canisius

Netherlands

Yiqun Cao

USA

John Capra

USA

Emidio Capriotti

USA

Albert Cardona

USA

Vincent Carey

USA

Ian Carr

UK

Joao Andre Carrico

Portugal

Antonio Carvajal-Rodriguez

Spain

Luis Carvalho

USA

Josep Casacuberta

Spain

Rita Casadio

Italy

Robert Castelo

Spain

Michele Ceccarelli

Italy

Gunnar Cedersund

Sweden

Turgay Celik

Singapore

Gustavo Cerqueira

USA

Luigi Cerulo

Italy

Monica Chagoyen

Spain

Yunrong Chai

USA

Sutirtha Chakraborty

USA

Robert Chalkley

USA

Hsueh-Wei Chang

Taiwan

Tien-Hao Chang

Taiwan

Claudine Chaouiya

Portugal

Wendy Chapman

USA

Sidhartha Chaudhury

USA

Chongjian Chen

China

Dung-Tsa Chen

USA

James Chen

USA

Liang Chen

USA

Mengjie Chen

USA

Peng Chen

Singapore

Rui Chen

USA

Wei Chen

Germany

Wei Chen

USA

Jianlin Cheng

USA

Li Cheng

Singapore

Lu Cheng

Finland

Divya Chhabra

USA

Derek Chiang

USA

Helene Chiapello

France

Matteo Chiara

Italy

Jeong-Hyeon Choi

USA

Nils Christian

UK

Jen-Hwa Chu

USA

Lisa Chung

USA

Ren-Hua Chung

Taiwan

Alexander Churbanov

USA

Piotr Cieplak

USA

Elisa Cilia

Italy

Jennifer Clarke

USA

Vincent Claveau

France

Lieven Clement

Belgium

Lieven Clement

Belgium

G. Marius Clore

USA

Peter Clote

USA

Luis Pedro Coelho

Germany

Kevin Cohen

USA

James Cole

USA

Jacques Colinge

Austria

Carlo Combi

Italy

Ana Conesa

Spain

Lee Cooper

USA

Davide Cora'

Italy

Heather Cordell

UK

Francesca Cordero

Italy

Ludovic Cottret

France

Francisco Couto

Portugal

Lindsay Cowell

USA

Cymon John Cox

Portugal

Engin Cukuroglu

Turkey

Alexandre Cunha

USA

Michal Dabrowski

Poland

Olivier Dameron

France

Theodoros Damoulas

USA

Thomas Dandekar

Germany

Sara D'Angelo

USA

Ludmila Danilova

USA

Michael Dark

USA

Heran Darwin

USA

Rhiju Das

USA

Sean Daugherty

USA

George Davey Smith

UK

Mariza De Andrade

USA

Riccardo De Bin

Germany

Pieter De Bleser

Belgium

Daniela De Canditiis

Italy

Xavier De La Cruz

Spain

Alberto De La Fuente

Germany

Sjoerd De Vries

Germany

Eric Debreuve

France

Frank Dehne

Canada

Yves Dehouck

Belgium

Nicolas Delhomme

Sweden

Nicolas Delhomme

Sweden

Jingyuan Deng

USA

Minghua Deng

China

James Dennis

Canada

Sebastian Deorowicz

Poland

Narayan Desai

USA

Christophe Dessimoz

UK

Colin Dewey

USA

Jo Dicks

UK

Shuyan Ding

China

Valentin Dinu

USA

R.W. Doerge

USA

Gunay Dogan

USA

Marlies Dolezal

Austria

Eytan Domany

Israel

Francisco Domingues

Italy

Ian John Donaldson

UK

Ruslan Dorfman

Canada

Edward Dougherty

USA

Robin Dowell

USA

Andreas Dräger

USA

Finn Drabløs

Norway

Sorin Draghici

USA

Yotam Drier

Israel

Amy Driskell

USA

Tom Druet

Belgium

Fang Du

China

Junbo Duan

USA

Joel Dudley

USA

Michel Dumontier

Canada

David Duverle

Japan

Oleh Dzyubachyk

Netherlands

Ingo Ebersberger

Germany

Bettina Ebert

Germany

Afshin Ebrahimpour

Malaysia

Kristin Eckel-Mahan

USA

Gerhard Ecker

Austria

Todd Edwards

USA

Guy Eelen

Belgium

Mikel Egana Aranguren

Spain

Michael Eiden

UK

Jens Einloft

Germany

Frank Eisenhaber

Singapore

Mohamed Elati

France

Nadia El-Mabrouk

Canada

Arne Elofsson

Sweden

Arne Elofsson

Dominica

Richard Emes

UK

Frank Emmert-Streib

UK

Fabian Erdel

Germany

Patricia Evans

Canada

Ludger Evers

UK

Konstantinos Exarchos

Greece

Daniel Falush

UK

Laura Fancello

France

David Fardo

USA

Piero Fariselli

Italy

Mario Fasold

Germany

Justin Fay

USA

Tom Fearn

UK

Nikolas Fechner

Germany

Dmitry Fedorov

USA

K. Anton Feenstra

Netherlands

Kurt Fellenberg

Germany

Xin Feng

USA

Malcolm Ferguson-Smith

UK

Michael Fernandez

Canada

Xose Fernandez

UK

Fulvia Ferrazzi

Germany

Andrew Filby

UK

Francesco Filippini

Italy

Nicolas Fiorini

France

Andras Fiser

USA

Danyel Fisher

USA

Jean-Pierre Flandrois

France

Ralf Floca

Germany

Darren Flower

UK

Stephen Fong

USA

Simon Forbes

UK

Dimitrios Fotiadis

Greece

Giuditta Franco

Italy

Lude Franke

Netherlands

David Fredman

Austria

Joel Freundlich

USA

Erwin Frise

USA

Martin Frith

Japan

Holger Fröhlich

Germany

Kimon Frousios

UK

Bo Fu

Canada

Yan Fu

China

Edvin Fuglebakk

Norway

Christopher Funk

USA

Cesare Furlanello

Italy

Laura Ines Furlong

Spain

Matthias Erwin Futschik

Portugal

Antonella Galli

UK

Giuseppe Gallone

UK

Michael Galperin

USA

Oxana Galzitskaya

Russian Federation

Chun-Fang Gao

China

Jun Gao

China

Pei Gao

UK

Vincent Gardeux

USA

Daniel Garrigan

USA

Pascale Gaudet

Switzerland

Huanying Ge

USA

Donald Geman

USA

George Georgakilas

Greece

Benjamin Georgi

USA

Samuel Gerber

USA

Wolfgang Gerlach

USA

Jana Gevertz

USA

Debashis Ghosh

USA

Pratim Ghosh

USA

Yolanda Gil

USA

Walter Gilks

UK

Filip Ginter

Finland

Charles Girardot

Germany

Enrico Glaab

Luxembourg

Daniel Glez-Peña

Spain

William Godinez Navarro

Germany

Carsten Goerg

USA

Tatyana Goldberg

Germany

Benjamin Goldstein

USA

F. Xavier Gomis-Rüth

Spain

Faviel Gonzalez Galarza

UK

Benjamin Good

USA

Leo Goodstadt

UK

Balasubramanian Gopal

India

Derek Gordon

USA

Pawel Gorecki

Poland

Johan Goris

Belgium

Valer Gotea

USA

Susumu Goto

Japan

Julian Gough

UK

Joerg Graf

USA

Trevor Graham

UK

Urs Greber

Switzerland

Casey Greene

USA

Russell Greiner

Canada

Sam Griffiths-Jones

UK

Hinrich Gronemeyer

France

Shari Grossman

USA

Georg Groth

Germany

Mathieu Groussin

France

Aleksandra Gruca

Poland

Sergei Grudinin

France

Christoph Grunau

France

Yongtao Guan

USA

Fabrizio Guarneri

Italy

Mario Rosario Guarracino

Italy

Concettina Guerra

USA

Rudy Guerra

USA

Vincent Guillemot

France

Rudiyanto Gunawan

Switzerland

Cigdem Gunduz-Demir

Turkey

Jun-Tao Guo

USA

Maozu Guo

China

Wei Guo

USA

Weilong Guo

China

Tsuyoshi Hachiya

Japan

Amber Hackstadt

USA

Jonathan Haines

USA

Iman Hajirasouliha

USA

Iman Hajirasouliha

Canada

Marc Halfon

USA

Brian Halligan

USA

Michael Halter

USA

Michiaki Hamada

Japan

Nicholas Hamilton

Australia

Kim Hammond-Kosack

UK

Fred Hamprecht

Germany

Jing-Dong Han

China

Leng Han

USA

Lianyi Han

USA

Xiaoxu Han

USA

Kousuke Hanada

Japan

Sridhar Hannenhalli

USA

Kasper Hansen

USA

Ulrich Hansmann

USA

Israel Hanukoglu

Israel

Jin-Kao Hao

France

Tong Hao

China

Alexander Hapfelmeier

Germany

Thomas James Hardcastle

UK

Henk Harkema

UK

Dag Harmsen

Germany

Brigitte Hartmann

France

Michael Hartung

Germany

Bernhard Haubold

Germany

Michael Hawrylycz

USA

Steven Hayward

UK

Lenwood Heath

USA

Lenwood Heath

USA

Tim Hefferon

USA

Dominik Heider

Germany

Volkhard Helms

Germany

Alex Henneman

Netherlands

Jean-Karim Heriche

Germany

Jaap Heringa

Netherlands

David Hernandez

Switzerland

Carl Herrmann

France

Hanspeter Herzel

Germany

Steffen Heyne

Germany

John Hickey

Australia

Yulia Hicks

UK

Des Higgins

Ireland

Andreas Hildebrandt

Germany

Steven Hill

UK

Blanca Himes

USA

Heinz Himmelbauer

Spain

Matthew Hindle

UK

Shivashankar Hiriyur Nagaraj

Australia

Shinn-Ying Ho

Taiwan

Simon Ho

Australia

Toby Dylan Hocking

France

Robert Hoehndorf

UK

Ralf Hofestädt

Germany

Michael Hoffman

Canada

Noah Hoffman

USA

Steve Hoffmann

Germany

Mark Holder

USA

Jim Holland

USA

Jaakko Hollmén

Finland

Arjen Hommersom

Netherlands

Antti Honkela

Finland

Farhad Hormozdiari

USA

Fereydoun Hormozdiari

Canada

David Horn

Israel

Dragos Horvath

France

Steve Horvath

USA

Lin Hou

USA

Yong Hou

China

E. Andres Houseman

USA

Valerie Hower

USA

William Hsiao

Canada

Sung-Tsang Hsieh

Taiwan

Li Hsu

USA

Gang Hu

China

Gangqing Hu

USA

Jianhua Hu

USA

Di Huang

USA

Fenix Huang

Denmark

Guohua Huang

China

Hsien-Da Huang

Taiwan

Weichun Huang

USA

Xueling Huang

China

Simon Hubbard

UK

Chad Huff

USA

Ken Hui

USA

Jui-Hung Hung

Taiwan

Hongwei Huo

China

Curtis Huttenhower

USA

Susan Idicula-Thomas

India

Chris Illingworth

UK

Hae Kyung Im

USA

Francesco Iorio

UK

Shumpei Ishikawa

Japan

Hemant Ishwaran

USA

Rezarta Islamaj Dogan

USA

Laurent Jacob

France

Samira Jaeger

Spain

Vignesh Jagadeesh

USA

Tobias Jakobi

Germany

Aruna Jammalamadaka

USA

Woncheol Jang

Korea

Vuk Janjic

UK

Jean-Luc Jannink

USA

Stefan Janssen

Germany

Nathalie Japkowicz

Canada

Khuloud Jaqaman

USA

Herbert Jelinek

Australia

Andrew Jenkinson

UK

Livnat Jerby

Israel

Hongkai Ji

USA

Shuiwang Ji

USA

Tieming Ji

USA

Dingfeng Jiang

USA

Ning Jiang

USA

Rui Jiang

China

Glen Jickling

USA

Antonio J Jimeno- Yepes

USA

Guangxu Jin

USA

Victor Jin

USA

Peter Johansson

Australia

Andrew Johnson

USA

Charles Johnson

USA

W. Evan Johnson

USA

Inge Jonassen

Norway

Martin Jones

UK

Siddhartha Jonnalagadda

USA

Fabrice Jossinet

France

Fabien Jourdan

France

Hsueh-Fen Juan

Taiwan

Klaus Jung

Germany

Astrid Junker

Germany

Elizabeth Jurrus

USA

Lars Kaderali

Germany

Koji Kadota

Japan

Tamer Kahveci

USA

Yannis Kalaidzidis

Germany

Alfredo Kalaitzis

UK

Christoph Kaleta

Germany

Olga Kalinina

Germany

Lee Kamentsky

USA

Zachary Kaminsky

USA

Michael Kane

USA

Haruhiko Kaneko

Japan

Dongwan Kang

USA

Kasthuri Kannan

USA

Wei-Chun Kao

Taiwan

Emre Karakoc

USA

Konrad Karczewski

USA

Wojciech Karlowski

Poland

Peter Karp

USA

Hisashi Kashima

Japan

Khalil Kashkush

Israel

Riku Katainen

Finland

Kazutaka Katoh

Japan

Lee Katz

USA

Michael Katze

USA

Enver Kayaaslan

Turkey

Dukka Kc

USA

Chen Keasar

Israel

Karen Keating

USA

Alexander Kel

Germany

Steven Kelk

Netherlands

Esther Kellenberger

France

Andrew Kernytsky

USA

Ozlem Keskin

Turkey

Juha Kesseli

Finland

Hector Keun

UK

Asifullah Khan

Pakistan

Warren Kibbe

USA

Marcin Kierczak

Sweden

Daisuke Kihara

USA

Jin-Dong Kim

Japan

Jung-Jae Kim

Singapore

Philip Kim

USA

Sun Kim

USA

Shuhei Kimura

Japan

Carleton Kingsford

USA

Martin Kircher

Germany

Martin Kircher

USA

Paul Kirk

UK

Hisanori Kiryu

Japan

James Kitchen

UK

Gunnar W. Klau

Netherlands

Bernd Klaus

Germany

Holger Klein

Germany

Konstantin Klenin

Germany

Kjetil Klepper

Norway

David Klinke

USA

Bernhard Knapp

Austria

David W. Knowles

USA

Dennis Ko

USA

Ina Koch

Germany

Hiroshi Kohsaka

Japan

Grigory Kolesov

USA

Ravikumar Komandur Elayavilli

USA

Yong Kong

USA

Frank Konietschke

Germany

Jan Korbel

Germany

Ian Korf

USA

Sophia Kossida

Greece

Mehmet Koyuturk

USA

Antje Krause

Germany

Rajanikant Krishnamurthy

India

Janos Kriston-Vizi

Singapore

Martin Krzywinski

Canada

Pei Fen Kuan

USA

Rui Kuang

USA

Ursula Kummer

Germany

Sudip Kundu

India

Hiroyuki Kurata

Japan

Nikos Kyrpides

USA

Yinglei Lai

USA

Philippe Laissue

UK

Tak-Wah Lam

Hong Kong

Teddy Lam

Hong Kong

Moritz Lang

Switzerland

Morgan Langille

USA

Jessica Larson

USA

Ines Latka

Germany

Augustinus Laude

Singapore

Arvydas Laurinavicius

Lithuania

Berthold Lausen

UK

Dominique Lavenier

France

Richard Lavery

France

Michael Lawrence

USA

Daniel Lawson

UK

Barbara Lazzari

Italy

Kim-Anh Lê Cao

Australia

Nicolas Le Novere

UK

Christopher Leckie

Australia

Chih Lee

USA

Gyemin Lee

Korea

Sanghyuk Lee

Korea

Seunghak Lee

USA

Seungyeoun Lee

Korea

Tzong-Yi Lee

Taiwan

Jeffrey Leek

USA

Elliot Lefkowitz

USA

Marie-Paule Lefranc

France

Kjong Lehmann

USA

Xiujuan Lei

China

Forian Leitner

Spain

Ney Lemke

Brazil

Marc Lensink

France

Guy Leonard

UK

Emmanuelle Lerat

France

Henry C.M. Leung

Hong Kong

Ks Leung

Hong Kong

Zoran Levnajic

Slovenia

Biao Li

USA

Bo Li

USA

Chen Li

China

Cong Li

USA

Gang Li

USA

Gengxin Li

USA

Guohui Li

China

Honglin Li

China

Jinyan Li

Australia

Jun Li

USA

Kejie Li

USA

Kelvin Li

USA

Miaoxin Li

Hong Kong

Min Li

China

Ming Li

USA

Qunhua Li

USA

Shaoyu Li

USA

Shuai Cheng Li

Canada

Shuaicheng Li

Hong Kong

Wei Li

USA

Weizhong Li

USA

Xiaohong Li

USA

Xin (James) Li

USA

Yang Li

USA

Yongjin Li

USA

Zhiguo Li

USA

Binhua Liang

Canada

Chun Liang

USA

Arthur Liberzon

USA

Jason Lieb

USA

Michael Liebling

USA

Sara Light

Sweden

Elaine Lim

USA

Zhixiang Lin

USA

Michael Linderman

USA

Matthew Links

Canada

Michael Liss

Germany

Ching-Ti Liu

USA

Dajiang Liu

USA

Feifan Liu

USA

Han Liu

USA

Hongfang Liu

USA

Jimin Liu

Singapore

Jin Liu

USA

Liang Liu

USA

Peng Liu

USA

Peng Liu

USA

Tao Liu

USA

Tianming Liu

USA

Wei Liu

UK

Xiao Liu

China

Xiaoming Liu

USA

Xiaowen Liu

USA

Yan Liu

USA

Yaping Liu

USA

Yin Liu

USA

Yongchao Liu

Germany

Yu-Shen Liu

China

Zhenqiu Liu

USA

Vebjorn Ljosa

USA

Nicholas Loman

UK

Stefano Lonardi

USA

Douglas Londono

USA

Fuhui Long

USA

Luca Longo

Ireland

Lit-Hsin Loo

Singapore

Hugo López Fernández

Spain

Belen Lorente-Galdos

Spain

Doug Lorenz

USA

Stephan Lorenzen

Germany

Justo Lorenzo Bermejo

Germany

Xinghua Lou

USA

Chuan Lu

UK

Xinghua Lu

USA

Yue Lu

USA

Zhi Lu

China

Zhiyong Lu

USA

Tu Luan

Norway

Steven Lund

USA

Gerton Lunter

UK

Jie Luo

USA

Lara Lusa

Slovenia

Jian Ma

USA

Shuangge Ma

USA

Kathy Macropol

USA

Paolo Magni

Italy

Eamonn Maguire

UK

Padmanabhan Mahadevan

USA

Shaun Mahony

USA

Noel Malod-Dognin

UK

John Manak

USA

Yael Mandel-Gutfreund

Israel

Bangalore Manjunath

USA

Jim Manos

Australia

Ulrich Mansmann

Germany

Yuqing Mao

USA

Daniel Marbach

USA

Bickle Marc

Germany

Guillaume Marcais

USA

Guillaume Marcais

USA

Sergio Marchini

Italy

Raphaël Marée

Belgium

Georgi Marinov

USA

John Marioni

UK

Guillemette Marot

France

Tobias Marschall

Netherlands

Pier Luigi Martelli

Italy

Lennart Martens

Belgium

Maria J. Martin

UK

Anna Maria Masci

USA

Andres R. Masegosa

Spain

Ali Masoudi-Nejad

Iran

Naim Matasci

USA

Catherine Mathé

France

Premendu P Mathur

India

Frederick Matsen

USA

Pär Matsson

Sweden

Georg Matuschek

Germany

Markus Maucher

Germany

Giancarlo Mauri

Italy

Raja Mazumder

USA

Matthew Nicholson Mccall

USA

Fiona Mccarthy

USA

Monnie Mcgee

USA

Paul Mcgettigan

Ireland

Lauren Mcintyre

USA

Steve Mckeever

Sweden

Geoff Mclachlan

Australia

Vannary Meas-Yedid

France

Chandrasekaran Meganathan

India

Francisco Melo

Chile

Vesna Memisevic

USA

Pedro Mendes

UK

Bjoern Menze

Switzerland

Ivan Merelli

Italy

Brian Metscher

Austria

Patrick Emmanuel Meyer

Belgium

Gos Micklem

UK

Jeffrey Miecznikowski

USA

Istvan Miklos

Hungary

Crispin Miller

UK

Ryan Mills

USA

Natasa Miskov-Zivanov

USA

Jennifer Mitchell

Canada

Julie Mitchell

USA

Abhijit Mitra

India

Riten Mitra

USA

Qianxing Mo

USA

Perry Moerland

Netherlands

Mohd Saberi Mohamad

Malaysia

Yassene Mohammed

Netherlands

Debasisa Mohanty

India

Rosma Mohd Dom

Malaysia

Elodie Monsellier

France

Catherine Mooney

Ireland

Jason Moore

USA

Patrick S. Moore

USA

Sandro Morganella

Italy

Giulia Morra

Italy

Kris Morreel

Belgium

John Morris

USA

Quaid Morris

Canada

Giulia Morsica

Italy

Charalampos Moschopoulos

Belgium

Vincent Moulton

UK

Raphael Mourad

USA

Simon Moxon

UK

Jan Mrazek

USA

Fabian Mueller

Germany

Jonathan W Mueller

UK

Jennifer Mulle

USA

Dragos Munteanu

USA

Usha Muppirala

USA

Chris Myers

USA

Seung Jin Na

USA

Jose Nacher

Japan

Radhakrishnan Nagarajan

USA

Masahiko Nakatsui

Japan

Dougu Nam

Korea

Loris Nanni

Italy

Chanin Nantasenamat

Thailand

Arunachalam Narayanaswamy

USA

Giuseppe Narzisi

USA

Alejandro Nato

USA

Armando Navarro-Vázquez

Spain

Hammad Naveed

Saudi Arabia

Dan Nettleton

USA

Pierre Neuvial

France

Bruno Nevado

Spain

Richard Newton

UK

Shu-Kay Ng

Australia

Thanh-Phuong Nguyen

Italy

Kristin Nicodemus

Ireland

Henrik Nielsen

Denmark

Stefan Niemann

Germany

Norbert Niklas

Austria

Kang Ning

China

Ken Nishikawa

Japan

Masha Niv

Israel

Fumio Nomura

Japan

Intawat Nookaew

Sweden

Michael North

USA

Natasha Noy

USA

Ibrahim Numanagic

Canada

Ruth Nussinov

USA

Tom Nye

UK

Mark Oakley

UK

Valerie Obenchain

USA

Michael Ochs

USA

Charles O'Donnell

USA

Hiroyuki Ogata

France

Marco Rinaldo Oggioni

Italy

Uwe Ohler

USA

Uwe Ohler

Germany

Mattias Ohlsson

Sweden

Constantin Orasan

UK

Nikita Orlov

USA

Raydonal Ospina

Brazil

Yu-Yen Ou

Taiwan

Zhengqing Ouyang

USA

Lex Overmars

Netherlands

Alexandra Pacureanu

Sweden

Dirk Padfield

USA

Luca Pagani

UK

Roderic Page

UK

Stefano Maria Pagnotta

Italy

Vasile Palade

UK

Leon Palafox

Japan

Xianming Pan

China

Vera Pancaldi

Spain

Anna Panchenko

USA

Gaurav Pandey

USA

Arun Prasad Pandurangan

UK

Herbert Pang

USA

Nikos Papandreou

Greenland

Balázs Papp

Hungary

Cheolwoo Park

USA

Heejin Park

Korea South

Sheldon Park

USA

Taesung Park

Korea South

Brian Parker

Australia

Amrita Pati

USA

Ellis Patrick

Australia

Rob Patro

USA

Gregory Paul

Switzerland

Paul Pavlidis

Canada

Stelios Pavlidis

UK

Georgios Pavlopoulos

Greece

Yudi Pawitan

Sweden

Philip Payne

USA

William Pearson

USA

Jean Peccoud

USA

Shyamal Peddada

USA

Christian Pedersen

Denmark

Jianfeng Pei

China

Hanchuan Peng

USA

Bethany Percha

USA

Miguel Perez-Enciso

Spain

Bengt Persson

Sweden

Catia Pesquita

Portugal

Britt-Sabina Petersen

Germany

Kjell Petersen

Norway

Jean-Baptiste Pettit

UK

Linda Petzold

USA

Ulrich Pfeffer

Italy

Nico Pfeifer

Germany

Ernesto Picardi

Italy

Alexander Pico

USA

William Piel

USA

Lindenbaum Pierre

France

Tobias Pietzsch

Germany

Roger Pique-Regi

USA

Rimma Pivovarov

USA

Vincent Plagnol

UK

Francisco Planes

Spain

Matthias Platzer

Germany

Dariusz Plewczynski

Poland

Laila Poisson

USA

Victor Pomareda

Spain

Julia Ponomarenko

USA

Mihai Pop

USA

Alexey Porollo

USA

Nader Pourmand

USA

Uberto Pozzoli

Italy

Snehit Prabhu

USA

Snehit Prabhu

USA

Sylvain Pradervand

Switzerland

Ashok Prasad

USA

Stephan Preibisch

Germany

Jaime Prilusky

Israel

Sebastian Proost

Belgium

Mattia Prosperi

USA

Kim Pruitt

USA

Teresa Przytycka

USA

Gordon D. Pusch

USA

Catherine Putonti

USA

Sampo Pyysalo

Japan

Ji Qi

China

Yanjun Qi

USA

Sanbo Qin

USA

Jian-Ding Qiu

China

Weiliang Qiu

USA

Xing Qiu

USA

Long Qu

USA

Melanie Quintana

USA

Predrag Radivojac

USA

Bernhard Radlwimmer

Germany

Mark Ragan

Australia

Gajendra Raghava

India

Emanuele Raineri

Spain

Nasir Rajpoot

UK

Sripad Ram

USA

Matteo Ramazzotti

Italy

Pauli Rämö

Switzerland

Bruce Rannala

USA

Josef Daniel Rasinger

Norway

Oliver Ratmann

UK

Andrea Rau

France

Matteo Re

Italy

Pedro Reche

Spain

Robert Reid

USA

Jaques Reifman

USA

Mark Reimers

USA

Gesine Reinert

UK

Caroline Relton

UK

Stefan Rensing

Germany

David Reshef

USA

Boris Reva

USA

Mina Rho

USA

Pedro Ribeiro

Portugal

William Ricke

USA

Pentti Tapio Riikonen

Finland

Davide Risso

USA

Marylyn Ritchie

USA

Alberto Riva

USA

Charles Robert

France

Adam Roberts

USA

Gordon Robertson

Canada

Ashley Robinson

USA

Mark Robinson

Switzerland

Andrei Rodin

USA

Ingo Roeder

Germany

Torbjorn Rognes

Norway

Gustavo Rohde

USA

Roberto Romeor

USA

Olaf Ronneberger

Germany

Peter Rose

USA

Gail Rosen

USA

Omar Rota-Stabelli

Italy

Eric Rouchka

USA

Juho Rousu

Finland

Sushmita Roy

USA

Jean-Francois Rual

USA

Mirabela Rusu

USA

Morten Rye

Norway

Paul Ryvkin

USA

Andrey Rzhetsky

USA

Simon Sadedin

Australia

Yvan Saeys

Belgium

Julio Saez-Rodriguez

UK

David Safranek

Czech Republic

Daniel Sage

Switzerland

Marie France Sagot

France

Milton Saier

USA

Kenji Saitoh

Japan

Manuel Salvadores

USA

Michael Sammeth

Spain

Aminael Sanchez-Rodriguez

Belgium

Clare Sansom

UK

Mihaela Sardiu

USA

Anindya Sarkar

USA

J Fah Sathirapongsasuti

USA

Jessica Satkoski Trask

USA

Kengo Sato

Japan

Eric Sayers

USA

Ivo Sbalzarini

Germany

Peter Scacheri

USA

Gerald Schaefer

UK

Alejandro Schaffer

USA

Leonard Schalkwyk

UK

Theresa Scharl

Austria

Robert Scharpf

USA

Michael G. Schimek

Austria

Alexander Schleiffer

Austria

Tamar Schlick

USA

Paul Schliekelman

USA

Alexander Schliep

USA

Alexander Schliep

UK

Ralf Schmid

UK

Bertil Schmidt

Singapore

Bertil Schmidt

Germany

Heiko Schmidt

Austria

Steffen Schober

Germany

Alexander Schoenhuth

Netherlands

Paul Schofield

UK

Ingrid Scholtens

Netherlands

Falk Schreiber

Germany

Martijn Schuemie

Netherlands

Marcel H. Schulz

Germany

Stefan Schulz

Germany

Benjamin Schuster-Böckler

UK

Russell Schwartz

USA

Celine Scornavacca

France

Michelle Scott

Canada

Alexander Sczyrba

Germany

Brian Searle

USA

Stefan Seemann

Germany

Mark Segal

USA

Nicola Segata

Italy

Nicola Segata

USA

Inseok Seo

USA

Selja Seppälä

USA

Nuno Sepulveda

UK

Oliver Serang

USA

Caraman Sergiu-Viorel

Romania

Bertrand Servin

France

Jun Sese

Japan

Venkatraman Seshan

USA

Joao Setubal

Brazil

Jahangheer Shaik

USA

Xueguang Shao

China

Itai Sharon

USA

Cody Sheik

USA

Hong-Bin Shen

China

Ronglai Shen

USA

Yufeng Shen

USA

Leming Shi

USA

Wei Shi

Australia

Tetsuo Shibuya

Japan

Daichi Shigemizu

Japan

Hiroshi Shimizu

Japan

Kana Shimizu

Japan

Kosaku Shinoda

USA

Maulik Shukla

USA

Jingna Si

China

Anne Siegel

France

Pedro Silva

Portugal

Noah Simon

USA

Richard Simon

USA

Thomas Simonson

France

Jared Simpson

Canada

Andrew Sims

UK

Shantanu Singh

USA

Christine Sinoquet

France

Sinosh Skariyachan

India

Pavel Skums

USA

André Skupin

USA

Rafael Slodzinski

Germany

Fatima Smagulova

France

Ihor Smal

Netherlands

Barry Smith

USA

Carol Soderlund

USA

Kaushal Solanki

USA

Imre Solti

USA

Chi Song

USA

Jiangning Song

Australia

Seongho Song

USA

Ramanathan Sowdhamini

India

Rainer Spang

Germany

Ola Spjuth

Sweden

George Spyrou

Greece

Narayanaswamy Srinivasan

India

Jason Stajich

USA

Daron Standley

Japan

Mario Stanke

Germany

Gerhard Steger

Germany

Oliver Stegle

Germany

Johannes Stephan

Germany

Ralf Steuer

Germany

John R. Stevens

USA

Gregor Stiglic

Slovenia

Chris Stoeckert

USA

Gustavo Stolovitzky

USA

Veronique Storme

Belgium

Marc Strickert

Germany

Korbinian Strimmer

Germany

Gang Su

USA

Zhen Su

China

Zhenqiang Su

USA

Senthil Subramanian

USA

Srikrishna Subramanian

India

Pavel Sumazin

USA

Changming Sun

Australia

Deqiang Sun

USA

Fengzhu Sun

USA

Lei Sun

Canada

Zhifu Sun

USA

Johan Suykens

Belgium

Baris Suzek

USA

Neil Swainston

UK

Michael Swartz

USA

Liskin Swint-Kruse

USA

Maciej Szymanski

Poland

Yuval Tabach

USA

David Tabb

USA

Leila Taher

Germany

Fariza Tahi

France

Takeyuki Tamura

Japan

Chuan-Yi Tang

Taiwan

Haixu Tang

USA

Hao Tang

USA

Kailin Tang

China

Kun Tang

China

Sonia Tarazona Campos

Spain

Gian Gaetano Tartaglia

Spain

Tolga Tasdizen

USA

Nicholas Tatonetti

USA

Vladimir Teif

Germany

Andrew Teschendorff

UK

Peter Tessier

USA

Mukund Thattai

India

Nathalie Theret

France

Kristof Theys

Belgium

Alun Thomas

USA

Julie Thompson

France

Robert Thomson

USA

Jian Tian

China

Tianhai Tian

Australia

Desiree Tillo

Canada

Andrew Titman

UK

Simon Tomlinson

UK

Xiaojun Tong

China

Prasad Tongaonkar

USA

Manabu Torii

USA

Silvio Tosatto

Italy

Nora Toussaint

USA

Zlatko Trajanoski

Austria

Achim Tresch

Germany

Tim Triche

USA

William Trimble

USA

Brett Trost

Canada

Richard Tzong-Han Tsai

Taiwan

Tsung-Heng Tsai

USA

George Tseng

USA

Allan Tucker

UK

Catalina Tudor

USA

Nurcan Tuncbag

USA

Chang-Shung Tung

USA

Chun-Wei Tung

Taiwan

Svitlana Tyekucheva

USA

Joshua Udall

USA

Mohammad Shorif Uddin

Bangladesh

Hiroki R Ueda

Japan

Takeaki Uno

Japan

David Ussery

USA

Filippo Utro

USA

Cedric Vaillant

France

Gabriel Valiente

Spain

Giorgio Valle

Italy

David Vallenet

France

Johan Vallon-Christersson

Sweden

Stefan Van Aelst

Belgium

Alena Van Bömmel

Germany

Bas Van Breukelen

Netherlands

Mark Van De Wiel

Netherlands

Stijn Van Dongen

UK

Sacha Van Hijum

Netherlands

Sofie Van Landeghem

Belgium

Peter Van Loo

Belgium

Erik Van Mulligen

Netherlands

Alexis Vandenbon

Japan

Fabio Vandin

USA

Daniel Vanek

Czech Republic

Raghavan Varadarajan

India

Luca Varani

Switzerland

Charles Vaske

USA

Alexei Vazquez

USA

Miguel Vazquez

Spain

Charles Vejnar

USA

Vishwesh Venkatraman

Norway

Sergio Verjovski-Almeida

Brazil

Karin Verspoor

Australia

Jean-Philippe Vert

France

Francesco Vezzi

Sweden

Anand Vidyashankar

USA

Martin Vingron

Germany

Francisco Vital-Lopez

USA

Andreas Vlachos

UK

Ioannis Vlachos

Greece

Arndt Von Haeseler

Austria

Thomas Vondriska

USA

Ellen Voorhees

USA

Bjoern Voss

Germany

Jack Vossen

Netherlands

Gert Vriend

Netherlands

Carolina Wählby

Sweden

Matthew Wakefield

Australia

Levi Waldron

USA

Alan Walker

UK

Anders Wallqvist

USA

Ian Walsh

Italy

W. David Walter

USA

Dirk Walther

Germany

Lin Wan

USA

Raymond Wan

Hong Kong

Xiang Wan

Hong Kong

Hsiuying Wang

Taiwan

Jinhua Wang

USA

Likun Wang

China

Ming Wang

USA

Penghao Wang

Australia

Shuang Wang

USA

Ting Wang

China

Wei Wang

USA

Wenhui Wang

China

Xi Wang

Australia

Xiaosheng Wang

USA

Xiaowo Wang

China

Xin Victoria Wang

USA

Yu-Ping Wang

USA

Zhanyong Wang

USA

Gaby Wangorsch

Germany

Jim Warwicker

UK

Martin Wasser

Singapore

Josh Waterfall

USA

Christina Weaver

USA

Chaochun Wei

China

Wu Wei

USA

Zhi Wei

USA

Martin Weigt

France

Martin Weigt

France

Xiaoquan Wen

USA

David Robert Westhead

UK

Travis Wheeler

USA

Trish Whetzel

USA

Duangdao Wichadakul

USA

Susann Wicke

Germany

Ralph Wiedmann

USA

Chalini Wijetunge

Australia

W. John Wilbur

USA

Mark Wilkinson

Spain

Sebastian Will

Germany

Sebastian Will

USA

Brian Willett

UK

Kelly Williams

USA

Rohan Williams

Singapore

Tiffani Williams

USA

Robert Williamson

USA

Ole Winther

Denmark

K. Dane Wittrup

USA

Jason Wong

Australia

Jason Wong

UK

Thomas Wong

Hong Kong

Wing-Cheong Wong

Singapore

Hollis Wright

USA

Cen Wu

USA

Feizhen Wu

China

Hao Wu

USA

Ling-Yun Wu

China

Michael Wu

USA

Rongling Wu

USA

Song Wu

USA

Steven Wu

USA

Zheyang Wu

USA

Qian Xiang

China

Shu Xiao

USA

Yi Xiao

China

Conghua Xie

China

Lei Xie

USA

Tatiana Xifara

USA

Hao Xiong

USA

Dong Xu

USA

Jinfeng Xu

USA

Rong Xu

USA

Ying Xu

USA

Zhenyu Xuan

USA

Yu Xue

China

Yasunori Yamamoto

Japan

Yoshihiro Yamanishi

Japan

Xiting Yan

USA

Can Yang

USA

Cheng-Hong Yang

Taiwan

Chris Yang

USA

Jean Yang

Australia

Jianji Yang

USA

Li Yang

China

Pengyi Yang

Australia

Xiao Yang

USA

Xiaohan Yang

USA

Xinan Yang

China

Chen Yanover

USA

Omer Nebil Yaveroglu

UK

Ning Ye

Singapore

Jon Yearsley

Ireland

Lana Yeganova

USA

Ka Yee Yeung

USA

Yiming Ying

UK

Illhoi Yoo

USA

Erdem Yoruk

USA

Guangchuang Yu

China

Guoqiang Yu

USA

Shipeng Yu

USA

Yang Yu

USA

Gen Hua Yue

Singapore

Eleni Zacharia

Greece

Osvaldo Zagordi

Switzerland

Hossein Zare

USA

Nela Zavaljevski

USA

Mihaela Zavolan

Switzerland

Riccardo Zecchina

Italy

Sonja Zehetmayer

Austria

Alex Zelikovsky

USA

Oliver Zerbe

Switzerland

Bo Zhang

USA

Dabing Zhang

China

Heping Zhang

USA

Jack Zhang

USA

Jing Zhang

USA

Kurt Zhang

USA

Li Zhang

USA

Qiangfeng Zhang

USA

Songmao Zhang

China

Xian Zhang

USA

Xiangde Zhang

China

Zhongqi Zhang

USA

Min Zhao

USA

Patrick Xuechun Zhao

USA

Xingming Zhao

China

Heping Zheng

USA

Huiru Zheng

UK

Jie Zheng

Singapore

Xiaobin Zheng

USA

Xiaojing Zheng

USA

Xiaoqi Zheng

USA

Degui Zhi

USA

Jianlong Zhou

Australia

Jiayu Zhou

USA

Jie Zhou

USA

Jun Zhou

Australia

Peng Zhou

China

Qing Zhou

USA

Yaoqi Zhou

USA

Zhi Zhou

USA

Dajiang Zhu

USA

Hongjian Zhu

USA

Piotr Zielenkiewicz

Poland

Mark Ziemannn

Australia

Or Zuk

USA

Markus Zweckstetter

Germany

